# Efficacy of the PainVision apparatus for assessment of axial neck pain after cervical laminoplasty: a prospective study

**DOI:** 10.1186/s13018-023-03904-4

**Published:** 2023-06-30

**Authors:** Takeshi Inoue, Shigeru Soshi, Shun Yamamoto, Mitsuru Saito

**Affiliations:** Department of Orthopaedic Surgery, The Jikei University School of Medicine, 3-25-8 Nishishimbashi, Minato-ku, Tokyo 105-8461 Japan

**Keywords:** Axial neck pain, Laminoplasty, Cervical myelopathy, Pain, Axial symptoms, PainVision

## Abstract

**Background:**

Axial neck pain is one of the complications of posterior cervical surgeries such as laminoplasty. This study aimed to investigate the efficiency of the PainVision apparatus for axial neck pain assessment by comparing it with other methods.

**Methods:**

This prospective study included 118 patients (90 men and 28 women; average age: 66.9 (32–86) years) with cervical myelopathy who underwent open-door laminoplasty at our medical center between April 2009 and August 2019. Pain degree (PD) measured by PainVision, visual analog scale (VAS), and bodily pain (BP), a subitem of the MOS 36-Item Short-Form Health Survey (SF36), were used to evaluate axial neck pain, which was investigated preoperatively and at 3, 6, 12, 18, and 24 months postoperatively.

**Results:**

Comparison of the scores at each evaluation time point found significant improvement between the pre- and post-operative values for all assessment methods. Further, on comparing the amounts of change between pre- and post-operative scores in each pain assessment method, we found significant differences in PD and VAS but not in BP. We also found significant positive correlations between PD and VAS at each time point (all *p* < 0.001) and significant negative correlations between PD and BP (all *p* < 0.05) and between VAS and BP (all *p* < 0.01)  at each time point.

**Conclusions:**

In this study, we demonstrated that PD and VAS are more sensitive indicators of changes in axial neck pain than BP and also that PD has an excellent correlation with VAS. These results suggest that the PainVision apparatus may be an effective instrument for quantifying axial neck pain after cervical laminoplasty, though its superiority over VAS needs to be verified in future studies.

## Background

Cervical myelopathy is a progressive degenerative disease that causes sensory disturbance in the extremities, dysfunction of hand movement, gait disturbance, and dysuria [1]. Laminoplasty was developed as a surgical method for cervical myelopathy and is widely performed. However, one of the complications of laminoplasty is neck and shoulder pain, referred to as axial symptoms. It is also defined as axial neck pain [2], which significantly affects quality of life [3]. It is reported that axial neck pain has improved due to various improvements in surgical methods, rehabilitation, and orthosis [4–7]; however, the evaluation criteria of axial neck pain vary across reports, and it is notoriously difficult to evaluate pain in an objective and robust manner. This is in part due to the wide variability in pain thresholds and pain tolerance between individuals.

The PainVision PS-2100 (Nipro, Osaka, Japan), a quantitative pain analyzer, has been clinically applied to low back pain disorders, herpes zoster, and cancer pain [8–10]. However, to the best of our knowledge, no study has reported on assessment of axial neck pain after cervical laminoplasty. Therefore, this study aimed to investigate the efficacy of the PainVision apparatus for axial neck pain assessment by comparing it with other assessment methods.

## Methods

### Study population

This prospective study included a total of 118 patients with cervical myelopathy (110 patients with cervical spondylotic myelopathy; 8 with ossification of the posterior longitudinal ligament; 90 men and 28 women, with an average age of 66.9 years (32–86 years)) who underwent open-door laminoplasty at our medical center between April 2009 and August 2019. The cervical vertebral level at which laminoplasty was performed was C3–6 in 17 patients, C3–7 in 8, C4–6 in 87, and C4–7 in 6.

Patients with a history of cervical spine surgery, cervical radiculopathy, and those who received spinal fusion surgery together with laminoplasty were excluded from this study. This study was approved by the Ethics Committee of the local university. All patients were given an overview of the study either orally or in writing, and informed consent was obtained.

### Surgical technique

**Fig. 1:**
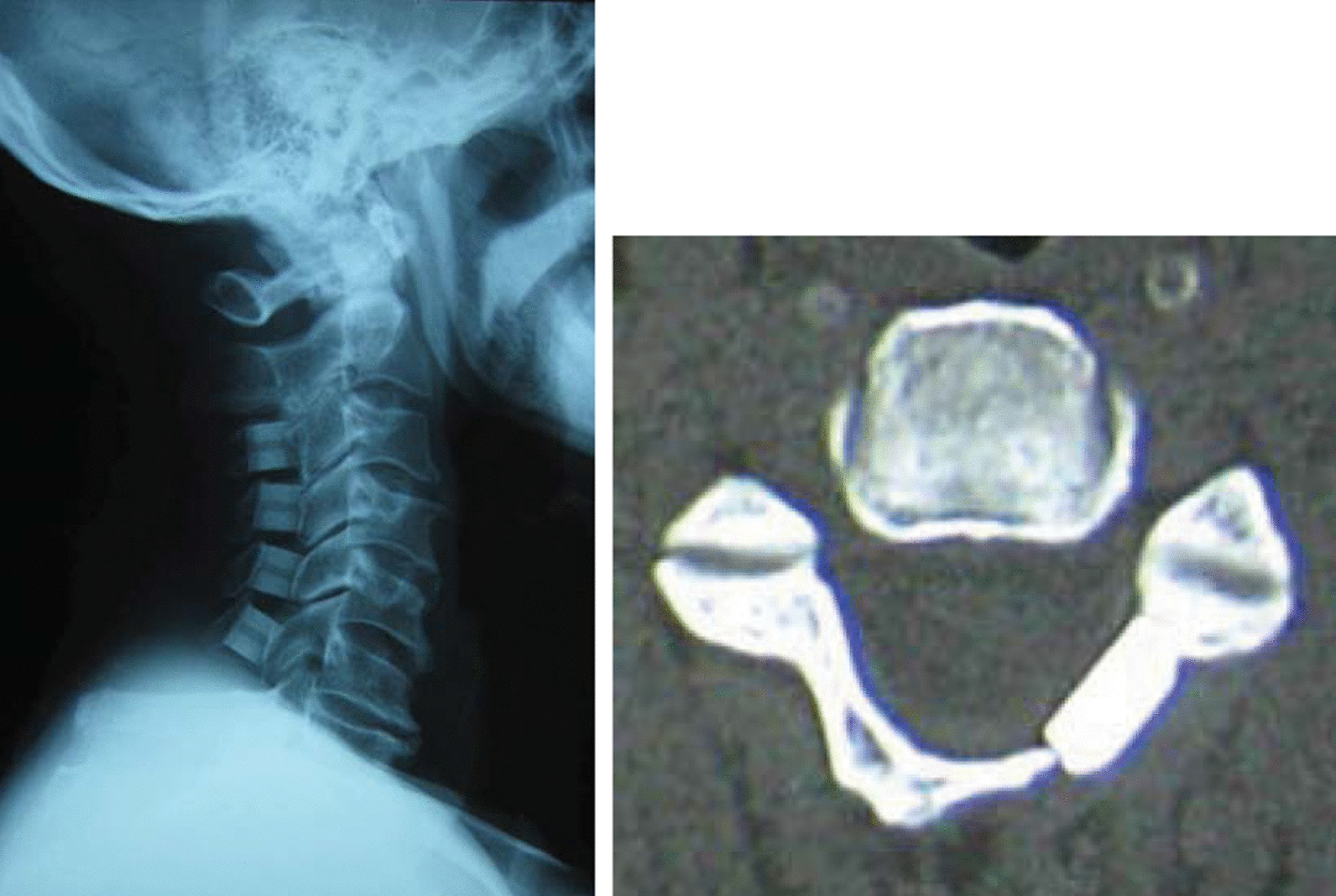
Post-operative X-rays and computed tomography images. Hydroxyapatite spacers were used for open-door laminoplasty in the present study

In this study, a single surgeon performed open-door laminoplasty using a slightly modified version of the Ito-Tsuji technique [7]. A posterior midline incision was performed, followed by an incision of the ligamentum nuchae. The detachment of muscle was performed slightly lateral to the lamina-facet junction. When the cervical vertebral levels at which laminoplasty was performed were C4–6, the muscles from the C3 to C6 were detached. However, the muscles attached to C2 and C7 were preserved. When the laminoplasty range included C3, the semispinalis cervicis muscle insertion at C2 was dissected. On the other hand, when the laminoplasty range included C7, the muscle attached to C7 was dissected. Gutters were fashioned on the inner edges of the facet joints on both the open and hinge sides. Following creation of the gutters, the lamina was opened and the ligamentum flavum as well as the epidural adhesion tissue on the open side was severed when necessary. Suture material was passed through a small burr hole that was created on the open side, and a hydroxyapatite spacer was placed to keep the lamina open (Fig. 1). A closed drain was inserted, and the wound was closed by suturing the ligamentum nuchae. On post-operative day 2, patients were allowed to leave their beds. Use of cervical collars was avoided where possible. During the early post-operative period, cervical spine range of motion training and isometric muscle strengthening were initiated.

### Outcome measures

**Fig. 2:**
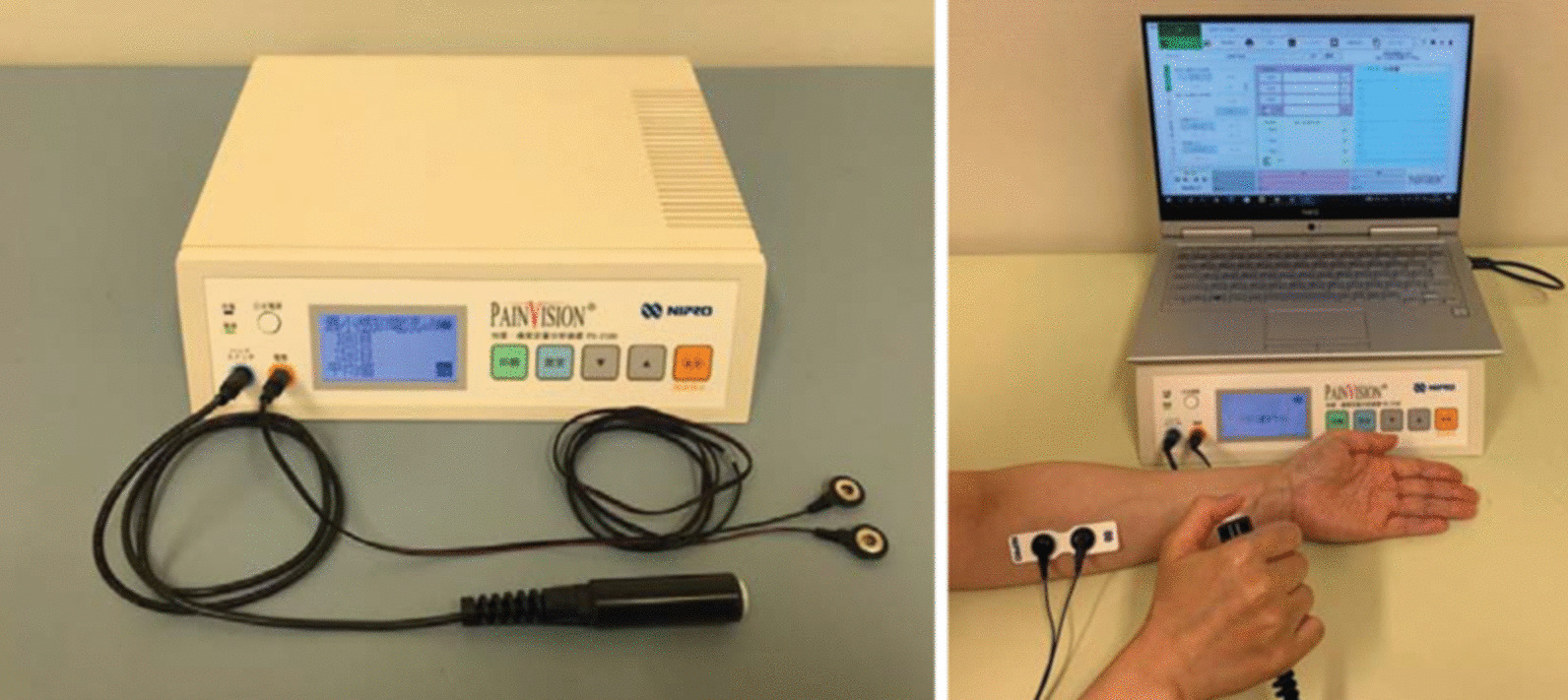
Photograph of the PainVision PS-2100 unit. The PainVision generated a superficial pulsed current that gradually increased in intensity and measured the participant’s perception thresholds and currently producing pain compatible with their axial neck pain were measured

The PainVision generated a superficial pulsed current (50 Hz; 0–150 μA; pulse amplitude, 0.3 ms) that gradually increased in intensity and measured the participant’s perception thresholds and current producing pain compatible with their axial neck pain (i.e., the current producing pain of the same intensity as the axial neck pain reported by the patient). We defined axial neck pain as pain or stiffness around the posterior neck or scapular areas in line with previous studies [4, 5]. The electrode of the PainVision was patched onto the surface of the patients’ forearm. The pain degree (PD) was automatically calculated (pain degree = 100 × (current producing pain comparable with axial neck pain—current at perception threshold) / current at perception threshold) (Fig. 2).

In addition to PD measured by the PainVision, visual analog scale (VAS) (score of 0 indicated no pain and 100 indicated worst pain) and bodily pain (BP) (the Japanese national standard was set at 50), a subitem of the MOS 36-Item Short-Form Health Survey (SF36), were used to evaluate axial neck pain and investigated preoperatively and at 3, 6, 12, 18, and 24 months postoperatively. The main analyses performed were as follows:

1. The axial neck pain scores preoperatively and at 3, 6, 12, 18, and 24 months postoperatively were compared using three pain assessment methods (PD, VAS, and BP).
2. The amounts of change between pre- and post-operative scores in three pain assessment methods were compared. The amount of change was defined as ( (scores at each assessment point) − (preoperative scores)) and evaluated at 3, 6, 12, 18, and 24 months postoperatively.
3. Correlation analyses between three types of pain assessment methods were evaluated.

### Statistical analysis

Statistical analyses were performed using SPSS, version 25.0 for Windows (IBM Japan, Tokyo, Japan). All values are expressed as mean ± standard deviation or as median and interquartile range (IQR). The Friedman test was used for intragroup comparisons after confirming the normality of the data beforehand. The Bonferroni method was used for post-tests. Spearman's rank correlation coefficient test was used for correlation analysis of the three pain assessment methods (PD, VAS, and BP). *P* values < 0.05 were considered statistically significant.

## Results

The median scores preoperatively and at 3, 6, 12, 18, and 24 months postoperatively in each assessment method were as follows (Table 1): PD, 82.5, 63.5, 52, 47, 43, and 31, respectively; VAS scores (mm), 43.5, 23.5, 20, 20, 20, and 16, respectively; and BP, 35.4, 40.3, 40.3, 40.3, 40.3, and 44.7, respectively.

**Table 1:** The pre- and post-operative scores in each pain assessment method and results of the Friedman test

		**Preop**	**3 months** **postop**	**6 months** **postop**	**12 months** **postop**	**18 months** **postop**	**24 months** **postop**	*p* **value**
**Pain degree** **(PD)**	Median (IQR)	82.5 (23.5–254.75)	63.5 (6.75–127.5)	52.0 (2–107)	47.0 (0–102.75)	43.0 (0–98.5)	31.0 (0–89)	*p* < 0.001
**VAS**	Median (IQR)	43.5 (10.25–68.75)	23.5 (8.5–47)	20.0 (7–49)	20.0 (5.25–45.75)	20.0 (0–43)	16.0 (0–33)	*p* < 0.001
**Bodily pain** **(BP)**	Median (IQR)	35.4 (26.9–44.7)	40.3 (35.4–50.1)	40.3 (35.4–50.1)	40.3 (35.4–49.9)	40.3 (35.4–50.1)	44.7 (35.4–54.6)	*p* < 0.001

All three pain assessment methods were found to be significantly improved postoperatively by the Friedman test

Preop., preoperatively; Postop., postoperatively; IQR, interquartile range; VAS, visual analog scale

### Comparison of the pre- and post-operative scores in each pain assessment method

Comparison of the pre- and post-operative scores using the Friedman test showed significant differences in all assessment methods (*p* < 0.001; Table 1). The post-test results using the Bonferroni method are shown in Table 2. For PD, significant differences were observed between scores preoperatively and 3 months postoperatively (*p* = 0.023), preoperatively and 6 months postoperatively (*p* = 0.004), preoperatively and 12 months postoperatively (*p* < 0.001), preoperatively and 18 months postoperatively (*p* < 0.001), and preoperatively and 24 months postoperatively (*p* < 0.001; Table 2). VAS scores showed significant differences between preoperatively and 12 months postoperatively (*p* = 0.013), preoperatively and 18 months postoperatively (*p* = 0.002), preoperatively and 24 months postoperatively (*p* < 0.001), and 6 months postoperatively and 24 months postoperatively (*p* = 0.024; Table 2). BP showed significant differences between preoperatively and 3 months postoperatively (*p* = 0.010), preoperatively and 6 months postoperatively (*p* = 0.005), preoperatively and 12 months postoperatively (*p* < 0.001), preoperatively and 18 months postoperatively (*p* < 0.001), and preoperatively and 24 months postoperatively (*p* < 0.001; Table 2).

**Table 2:** Comparison of pre- and post-operative scores in each pain assessment method using the Bonferroni method

PD**PD**	3 months	6 months	12 months	18 months	24 months
Preop	**0.023**	**0.004**	**0.000**	**0.000**	**0.000**
3 months		1.000	1.000	1.000	0.684
6 months			1.000	1.000	1.000
12 months				1.000	1.000
18 months					1.000

**VAS**	3 months	6 months	12 months	18 months	24 months
Preop	0.230	0.570	**0.013**	**0.002**	**0.000**
3 months		1.000	1.000	1.000	0.074
6 months			1.000	1.000	**0.024**
12 months				1.000	0.861
18 months					1.000

**BP**	3 months	6 months	12 months	18 months	24 months
Preop	**0.010**	**0.005**	**0.000**	**0.000**	**0.000**
3 months		1.000	1.000	1.000	0.290
6 months			1.000	1.000	0.480
12 months				1.000	1.000
18 months					1.000

Preop., preoperatively; PD, pain degree; VAS, visual analog scale; BP, bodily pain

### Comparison of the amounts of change between pre- and post-operative scores in each pain assessment method

Comparison of the amounts of change between pre- and post-operative scores using the Friedman test showed significant differences in PD and VAS scores but not in BP (Table 3). The results of the post-test using the Bonferroni method are shown in Table 4.

**Table 3:** Amounts of change between pre- and post-operative scores in each pain assessment method and results of the Friedman test

		**3 months** **postop**	**6 months** **postop**	**12 months** **postop**	**18 months** **postop**	**24 months** **postop**	*p* **value**
**Pain** **degree** **(PD)**	Median (IQR)	− 22.5 (− 154.25–3)	− 32 (− 151–0)	− 40.5 (− 179.5–21.5)	− 55 (− 172–0)	− 44.5 (− 172.5–0)	*p* = 0.002
**VAS**	Median (IQR)	− 6.5 (− 32.75–7.75)	− 6.5 (− 33.5–6.75)	− 10 (− 32.5–3.5)	− 11.5 (− 40–1.75)	− 11 (− 39–0)	*p* < 0.001
**Bodily pain** **(BP)**	Median (IQR)	4.5 (0–9.7)	4.7 (− 0.3–12.8)	4.9 (0–10.3)	4.5 (− 8.1–13.4)	5.4 (0–13.8)	*p* = 0.231

Comparison of the amounts of change between pre- and post-operative scores in each pain assessment method using the Friedman test showed significant differences in PD and VAS but not in BP

Postop., postoperatively; IQR, interquartile range; VAS, visual analog scale

**Table 4:** Comparison of the amounts of change between pre- and post-operative scores in each pain assessment method using the Bonferroni method

PD**PD**	6 months	12 months	18 months	24 months
3 months	1.000	0.530	**0.022**	**0.025**
6 months		1.000	0.323	0.358
12 months			1.000	1.000
18 months				1.000

**VAS**	6 months	12 months	18 months	24 months
3 months	1.000	1.000	0.055	**0.004**
6 months		1.000	0.248	**0.028**
12 months			1.000	0.262
18 months				1.000

**BP**	6 months	12 months	18 months	24 months
3 months				
6 months				
12 months				
18 months				

For BP, multiple comparisons were not performed because the Friedman test was not significant

PD, pain degree; VAS, visual analog scale; BP, bodily pain

For PD, a significant difference was noted between the amount of change at 3 months postoperatively and that at 18 months postoperatively (*p* = 0.022) and between that at 3 months postoperatively and that at 24 months postoperatively (*p* = 0.025; Table 4). For VAS scores, significant differences were observed at 3 months and 24 months postoperatively (*p* = 0.004) and at 6 months and 24 months postoperatively (*p* = 0.028; Table 4).

### Correlation analyses between the three pain assessment methods (PD, VAS, and BP)

Correlation analyses (Table 5) revealed significant positive correlations between PD and VAS scores at each time point (all *p* < 0.001) and significant negative correlations between PD and BP (all *p* < 0.05) and between VAS and BP at each time point (all *p* < 0.01).

**Table 5:** Correlation analyses between the three pain assessment methods (PD, VAS, and BP) using the Spearman's rank correlation coefficient test

**PD vs VAS**	PD Preop	PD 3 months	PD 6 months	PD 12 months	PD 18 months	PD 24 months
VAS Preop	*r* = 0.61 *p* < 0.001					
VAS 3 months		*r* = 0.63 *p* < 0.001				
VAS 6 months			*r* = 0.63 *p* < 0.001			
VAS 12 months				*r* = 0.79 *p* < 0.001		
VAS 18 months					*r* = 0.84 *p* < 0.001	
VAS 24 months						*r* = 0.85 *p* < 0.001

**PD vs BP**	PD Preop	PD 3 months	PD 6 months	PD 12 months	PD 18 months	PD 24 months
BP Preop	*r* = − 0.21 *p* = 0.02					
BP 3 months		*r* = − 0.40 *p* < 0.001				
BP 6 months			*r* = − 0.38 *p* < 0.001			
BP 12 months				*r* = − 0.37 *p* < 0.001		
BP 18 months					*r* = − 0.32 *p* = 0.001	
BP 24 months						*r* = − 0.30 *p* = 0.002

VAS vs BP	VAS Preop	VAS 3 months	VAS 6 months	VAS 12 months	VAS 18 months	VAS 24 months
BP Preop	*r* = − 0.29 *p* = 0.001					
BP 3 months		*r* = − 0.45 *p* < 0.001				
BP 6 months			*r* = − 0.47 *p* < 0.001			
BP 12 months				*r* = − 0.42 *p* < 0.001		
BP 18 months					*r* = − 0.34 *p* < 0.001	
BP 24 months						*r* = − 0.42 *p* < 0.001

Correlation analyses revealed significant positive correlations between PD and VAS at each time point (all *p* < 0.001), as well as significant negative correlations between PD and BP (all *p* < 0.05) and significant negative correlations between VAS and BP at each time point (all *p* < 0.01)

Preop., preoperatively; PD, pain degree; VAS, visual analog scale; BP, bodily pain

## Discussion

This study aimed to investigate the utility of PD, which was measured using the PainVision apparatus, as a pain assessment method by comparing it with VAS and BP. Through the prospective study of 118 patients with cervical myelopathy who underwent open-door laminoplasty at our medical center, we investigated axial neck pain preoperatively and at 3, 6, 12, 18, and 24 months postoperatively. We found PD and VAS to be more sensitive indicators of changes in axial neck pain than BP because comparing the amounts of change between pre- and post-operative scores in each pain assessment method showed significant differences in PD and VAS scores but not in BP. In addition, significant correlations were found in each pain assessment method, with robust positive correlations between PD and VAS.

Axial neck pain may increase after cervical posterior surgeries such as laminoplasty [11]. In a previous study, during the period when no preventive measures were taken, the incidence of axial pain after laminoplasty was 60%, and 25% of patients had severe pain that persisted for > 3 months after surgery [11]. In another study, laminoplasty and anterior cervical fusion were compared and the occurrence of axial pain after laminoplasty was found to be higher [12, 13].

Some preoperative factors and patient selection can influence outcomes following laminoplasty. For example, patients with a low mental state, potentially before surgery, are at a high risk of post-operative axial neck pain [14].

Many studies on preventive measures for axial neck pain have been reported [2, 4, 15–20]. A meta-analysis evaluating the presence of axial symptoms after posterior cervical decompression found that preservation of the posterior muscles and structures, stabilization of cervical vertebrae, and reduction in external cervical immobilization time were associated with reduced axial symptoms postoperatively [21]. The occurrence of axial neck pain, one of the complications after cervical laminoplasty, is likely to be decreasing due to the various modifications described above.

According to previous reports, different pain assessment methods were used for axial neck pain. There is no one method that can be termed the gold standard, probably because all methods have advantages and disadvantages. Hosono et al. [15] used a self-designed pain assessment method, wherein axial neck pain was graded according to previously published criteria, as follows: severe (pain killer or local injection regularly required), moderate (physiotherapy or compress regularly required), or mild (no treatment needed). Sakaura et al. also used this grading system [22], which is limited by the fact that the type and frequency of pain medications used varied between each patient. In a study by Mori et al. [16], VAS grades were used (I–IV): Grade I, 0–2.5 points; Grade II, 2.6–5.0 points; Grade III, 5.1–7.5 points; and Grade IV, 7.6–10.0 points, also limited by the arbitrary and artificial grade groupings. Takeuchi et al. described a self-designed pain assessment tool [4], which was graded as follows: no axial symptoms (no symptoms), occasional symptoms but no problems in activities of daily living (mild symptoms), and some problems in activities of daily living or work due to the symptoms (severe symptoms); however, the words ‘occasional’ and ‘some’ used in the gradings are subjective and open to differences in interpretation between individuals; for example, ‘occasional’ for one person will not be same as for another. Kimura et al. [3] rated axial neck pain on an 11-point scale from 0 to 10 using a numeric rating scale, and Yoshida et al. [6] described a self-designed pain assessment method graded 0–V: grade 0 = never, grade I = rarely, grade II = mild, grade III = moderate, grade IV = severe, and grade V = intolerable; this latter scale is also limited by the lack of precision in what would constitute ‘rarely,’ ‘mild,’ ‘moderate,’ ‘severe,’ and ‘intolerable,’ which would differ between individuals.

Pain assessment is essential for determining the effectiveness of treatment, and a method with scientific reliability and validity is ideal. However, pain is a purely subjective complaint and is impossible to evaluate in a truly objective manner. To this end, the goal should be the development of reliable and reproducible assessments of pain. In this study, three pain assessment methods, PD, VAS, and BP, were selected, and no obvious difference was noted in the sensitivity of the three methods when comparing scores at the time of each assessment. However, on comparing the change in scores of each evaluation method, significant differences were found in PD and VAS but not in BP. These results suggest that PD and VAS are more sensitive indicators of changes in axial neck pain than BP. It was difficult to establish relative superiority between PD and VAS. However, although VAS is useful for evaluating pain before and after treatment in the same case, it is difficult to use it to compare pain across cases [23]. On the other hand, PD is an evaluation method that applies perception thresholds; hence, there is a possibility that pain can be compared across cases [23]. Therefore, PD may be the preferred technique for studies comparing pain between different populations. VAS is also limited by ceiling effects that restrict the ability of patients to quantify worsening pain reliably [24] and conceal variation in the intensity of severe pain [25]; more specifically, the ceiling effect results in compression of all intensity ratings [26], and VAS anchors are poorly defined, thereby increasing the risk of misinterpretation, bias, and confusion [27].

PD has been measured using the PainVision in patients with low back pain previously, showing consistency with repeated calculations [23]. In another study that assessed low back pain, PD also showed a moderate correlation with the numeric rating scale scores at each time point [9]. Moreover, a previous study showed that the PainVision was also useful for the quantitative assessment of sensory disturbance [28, 29].

This study has some limitations. First, the PainVision is expensive (approximately 1,500,000 JPY or 11,500 USD) that hinders widespread use, particularly in resource-limited settings. Second, the lack of evaluation within the first 3 months following surgery. This was due to the fact that most patients in our medical center are transferred to other hospitals for rehabilitation after surgery.

## Conclusions

In this study, we demonstrated that PD and VAS are more sensitive indicators of changes in axial neck pain than BP and also that PD has an excellent correlation with VAS. These results suggest that the PainVision apparatus may be an effective instrument for quantifying axial neck pain after cervical laminoplasty, though its superiority over VAS needs to be verified in future studies.

## Data Availability

The datasets used and/or analyzed during the current study are available from the corresponding author on reasonable request.

## Abbreviations

PD: Pain degree
BP: Bodily pain
VAS: Visual analog scale
IQR: Interquartile range
