# Correction: Efficacy of the PainVision apparatus for assessment of axial neck pain after cervical laminoplasty: a prospective study

**DOI:** 10.1186/s13018-023-04277-4

**Published:** 2023-10-24

**Authors:** Takeshi Inoue, Shigeru Soshi, Shun Yamamoto, Mitsuru Saito

**Affiliations:** Department of Orthopaedic Surgery, The Jikei University School of Medicine, 3-25-8 Nishishimbashi, Minato-ku, Tokyo, 105-8461 Japan

**Correction: Journal of Orthopaedic Surgery and Research (2023) 18:473** 10.1186/s13018-023-03904-4

Following publication of the original article [1], the authors identified an error in Tables 1, 2, 4, and 5. The corrected figures are given below.

**Table 1:** The pre- and post-operative scores in each pain assessment method and results of the Friedman test

		Preop.	3 months postop.	6 months postop.	12 months postop.	18 months postop.	24 months postop.	p-value
Pain degree (PD)	Median (IQR)	82.5 (23.5–254.75)	63.5 (6.75–127.5)	52.0 (2–107)	47.0 (0–102.75)	43.0 (0–98.5)	31.0 (0–89)	p < 0.001
VAS	Median (IQR)	43.5 (10.25–68.75)	23.5 (8.5–47)	20.0 (7–49)	20.0 (5.25–45.75)	20.0 (0–43)	16.0 (0–33)	p < 0.001
Bodily pain (BP)	Median (IQR)	35.4 (26.9–44.7)	40.3 (35.4–50.1)	40.3 (35.4–50.1)	40.3 (35.4–49.9)	40.3 (35.4–50.1)	44.7 (35.4–54.6)	p < 0.001

All three pain assessment methods were found to be significantly improved postoperatively by the Friedman test

*Preop.* Preoperatively, *Postop.* Postoperatively, *IQR* interquartile range, *VAS* visual analogue scale

**Table 2:** Comparison of pre- and post-operative scores in each pain assessment method using the Bonferroni method

	3 months	6 months	12 months	18 months	24 months
*PD*					
Preop.	**0.023**	**0.004**	**0.000**	**0.000**	**0.000**
3 months		1.000	1.000	1.000	0.684
6 months			1.000	1.000	1.000
12 months				1.000	1.000
18 months					1.000
*VAS*					
Preop.	0.230	0.570	**0.013**	**0.002**	**0.000**
3 months		1.000	1.000	1.000	0.074
6 months			1.000	1.000	**0.024**
12 months				1.000	0.861
18 months					1.000
*BP*					
Preop.	**0.010**	**0.005**	**0.000**	**0.000**	**0.000**
3 months		1.000	1.000	1.000	0.290
6 months			1.000	1.000	0.480
12 months				1.000	1.000
18 months					1.000

*Preop.* Preoperatively, *PD* pain degree, *VAS* visual analogue scale, *BP* bodily pain

**Table 4:** Comparison of the amounts of change between pre- and post-operative scores in each pain assessment method using the Bonferroni method

	6 months	12 months	18 months	24 months
*PD*				
3 months	1.000	0.530	**0.022**	**0.025**
6 months		1.000	0.323	0.358
12 months			1.000	1.000
18 months				1.000
*VAS*				
3 months	1.000	1.000	0.055	**0.004**
6 months		1.000	0.248	**0.028**
12 months			1.000	0.262
18 months				1.000
*BP*				
3 months				
6 months				
12 months				
18 months				

For BP, multiple comparisons were not performed because the Friedman test was not significant

*PD* pain degree, *VAS* visual analogue scale, *BP* bodily pain

**Table 5:** Correlation analyses between the three pain assessment methods (PD, VAS, and BP) using the Spearman's rank correlation coefficient test

	PD Preop.	PD 3 months	PD 6 months	PD 12 months	PD 18 months	PD 24 months
*PD vs VAS*						
VAS Preop.	**r = 0.61** **p < 0.001**					
VAS 3 months		**r = 0.63** **p < 0.001**				
VAS 6 months			**r = 0.63** **p < 0.001**			
VAS 12 months				**r = 0.79** **p < 0.001**		
VAS 18 months					**r = 0.84** **p < 0.001**	
VAS 24 months						**r = 0.85** **p < 0.001**
*PD vs BP*						
BP Preop.	**r = −0.21** **p = 0.02**					
BP 3 months		**r = −0.40** **p < 0.001**				
BP 6 months			**r = −0.38** **p < 0.001**			
BP 12 months				**r = −0.37** **p < 0.001**		
BP 18 months					**r = −0.32** **p = 0.001**	
BP 24 months						**r = −0.30** **p = 0.002**

	VAS Preop.	VAS 3 months	VAS 6 months	VAS 12 months	VAS 18 months	VAS 24 months
*VAS vs BP*						
BP Preop.	**r = −0.29** **p = 0.001**					
BP 3 months		**r = −0.45** **p < 0.001**				
BP 6 months			**r = −0.47** **p < 0.001**			
BP 12 months				**r = −0.42** **p < 0.001**		
BP 18 months					**r = −0.34** **p < 0.001**	
BP 24 months						**r = −0.42** **p < 0.001**

Correlation analyses revealed significant positive correlations between PD and VAS at each time point (all p < 0.001), as well as significant negative correlations between PD and BP (all p < 0.05) and significant negative correlations between VAS and BP at each time point (all p < 0.01).

*Preop.* Preoperatively, *PD* pain degree, *VAS* visual analogue scale, *BP* bodily pain

The authors apologise for this error.

The original article [1] has been corrected.
